# Year-round dynamics of arbuscular mycorrhizal fungi communities in the roots and surrounding soils of *Cryptomeria japonica*

**DOI:** 10.1007/s00572-024-01143-x

**Published:** 2024-03-20

**Authors:** Akotchiffor Kevin Geoffroy Djotan, Norihisa Matsushita, Kenji Fukuda

**Affiliations:** Graduate School of Agricultural and Life Sciences (Laboratory of Forest Botany), University of Tokyo, 1-1-1, Yayoi, Bunkyo, Tokyo, 113-8657 Japan

**Keywords:** Forest ecosystem, AMF community, Seasonal dynamics, Soil properties, Microbial ecology, Next generation sequencing

## Abstract

Arbuscular mycorrhizal fungi (AMF) live simultaneously inside and outside of host plant roots for a functional mycorrhizal symbiosis. Still, the year-round dynamics and relationships between soil properties and AMF communities of trees in forest ecosystems remain unclear. We collected paired root and soil samples of the same *Cryptomeria japonica* trees at two forest sites (five trees at each site) every 2 months over a year. Total DNA was extracted from roots and soil separately and soil physicochemical properties were measured. With Illumina’s next-generation amplicon sequencing targeting the small subunit of fungal ribosomal DNA, we clarified seasonal dynamics of soil properties and AMF communities. Soil pH and total phosphorus showed significant seasonality while total carbon, nitrogen, and C/N did not. Only pH was a good predictor of the composition and dynamics of the AMF community. The total AMF community (roots + soil) showed significant seasonality because of variation from May to September. Root and soil AMF communities were steady year-round, however, with similar species richness but contained significantly different AMF assemblages in any sampling month. Despite the weak seasonality in the communities, the top two dominant OTUs showed significant but different shifts between roots and soils across seasons with strong antagonistic relationships. In conclusion, few dominant AMF taxa are dynamically shifting between the roots and soils of *C. japonica* to respond to seasonal and phenological variations in their microhabitats. AMF inhabiting forest ecosystems may have high environmental plasticity to sustain a functional symbiosis regardless of seasonal variations that occur in the soil.

**Supplementary Information:**

The online version contains supplementary material available at 10.1007/s00572-024-01143-x.

## Introduction

Arbuscular mycorrhizal fungi (AMF) live simultaneously inside and outside of host plant roots to enable functional mycorrhizal symbiosis. In some studies of AMF in plants, the roots had higher operational taxonomic unit (OTU) richness than the soil (Wang et al. 2015; Berruti et al. 2017; Mahmoudi et al. 2019) or vice versa (Xu et al. 2017; Faggioli et al. 2019; Gu et al. 2022; Faghihinia et al. 2022). These previous studies provide insights into the OTU richness of AMF communities in roots and soil but cannot further our understanding of AMF community dynamics in the roots and surrounding soils of host plants, particularly because most were single-sampling studies carried out independently in different environments using different methodologies. A few studies have attempted to clarify the seasonal or temporal variations in AMF communities, one in the roots and soils of three crop plants (Bainard et al. 2014) and the others in root fragments retrieved from soil cores collected in a grassland (Dumbrell et al. 2011; Barnes et al. 2016). While they detected seasonality and shifts of some AMF taxa, the studies either covered less than a full year or only investigated root AMF communities. Due to the two-habitat occupancy of AMF, investigations of their community dynamics should focus on both root and soil compartments while targeting a specific host plant. At present, the year-round dynamics and relationships between the root and soil AMF communities of a host plant remain unclear.

In forest ecosystems, variations in abiotic soil conditions, which are themselves determined by heterogeneous environmental conditions (Bahram et al. 2015), lead to fluctuations in soil microbial communities including AMF (Vályi et al. 2016; Nacke et al. 2016), which also is reported to be influenced by the species composition of the plant community (Grünfeld et al. 2021) and the spatial distribution of the host plants (Grünfeld et al. 2022). It is well established that abiotic factors such as soil physicochemical properties and climatic conditions significantly impact the development of AMF in the soil (Dumbrell et al. 2011; Jamiołkowska et al. 2018). Because of their two-habitat occupancy, the impact of soil physicochemical properties on the soil AMF may also reflect the impact on roots. While studies have reported that the root and soil AMF communities of a host plant respond differently to variations in soil physicochemical properties such as pH (Stevens et al. 2020; Djotan et al. 2023), little is known about how the seasonal variations in these soil physicochemical properties affect root and soil AMF communities. Therefore, the seasonal shifts in AMF taxa in response to soil temperature and physicochemical properties remain major gaps in our understanding of the ecology and biology of AMF.

*Cryptomeria japonica* (Cupressaceae) is an economically and ornamentally important tree in Japan, where it is the most widely planted species (Forestry Agency 2023). While studies have investigated its associated AMF communities (Matsuda et al. 2021; Djotan et al. 2022, 2023), the ecology of these communities remains unclear. The OTU richness of the AMF associated with *C. japonica* was lower in the roots than in the surrounding soil in winter (Djotan et al. 2022) but higher in the roots than in the soil in summer (Djotan et al. 2023). Other studies have reported seasonal dynamics of the fine root system of *C. japonica* (Konôpka et al. 2006; Tawa and Takeda 2016), and the AMF colonization of *C. japonica* roots and spore density in forest soil also show seasonality (Hata et al. 2018, 2023). Taken together, these findings suggest seasonal variation in the root and soil AMF communities of *C. japonica*. However, the seasonal dynamics and relationships between soil properties and the root and soil AMF communities of *C. japonica* remain uninvestigated.

In this study, we clarified the seasonal shifts of AMF taxa between roots and surrounding soils of *C. japonica*, evaluated the seasonal variation in soil physicochemical properties, and tested their year-round covariation. The same individual trees of *C. japonica* were sampled every 2 months from May 2021 to March 2022. Based on previous findings (Konôpka et al. 2006; Dumbrell et al. 2011; Bainard et al. 2014; Tawa and Takeda 2016; Hata et al. 2018, 2023; Djotan et al. 2022, 2023), we expected significant shifts within and between root and soil AMF communities of *C. japonica* across seasons under the influence of variations in soil physicochemical properties.

## Materials and methods

### Study sites and sampling design

This study was conducted at the University of Tokyo Chiba (UTCBF, 35.164 N latitude - 140.144 E longitude, 300 m a.s.l.) and Chichibu (UTCF, 35.944 N latitude - 138.824 E longitude, 1050 m a.s.l.) Forests (Online Resource 1). UTCBF and UTCF are located on steep slopes, where the plantations of *C. japonica* were established in 1927 (UTCBF) and 1980 (UTCF); the stand density was 600 and 1050 trees/ha at UTCBF and UTCF, respectively, but the understory of UTCBF was covered with many shrubs and herbaceous plants while that of UTCF had few plants (Djotan et al. 2023). The diameters at breast height of *C. japonica* trees were 49.4 ± 9.0 (UTCBF) and 32.6 ± 7.5 cm (UTCF). The environmental conditions at the sites during the sampling periods are presented in Table S1. At each site, we collected paired root and soil samples of the same five *C. japonica* trees every two months from May 2021 to March 2022 (Online Resource 2, Table 1). The investigated trees of *C. japonica* were distributed in an area of 1200 m^2^ (30 m × 40 m) in each of the study forests.

The sampling method described in Djotan et al. (2022) was employed. Briefly, rather than using soil coring, we tracked roots from the base of each targeted tree of *C. japonica*, cut them with scissors, and collected them together with the immediately surrounding soil (covering the roots as a buffer). At each sampling, roots were collected from a different side of the trees. Samplings in May, July, September, and November took place at the south, north, west, and east side of the tree, respectively. Samplings in January and March took place randomly around the target trees (not on a fixed side for all trees) but not at the previously sampled sides. At UTCF, sampling was not carried out in January 2022 and samples collected in July and September 2021 were excluded to maintain five biological replicates (number of trees = 5) for comparison between sampling months (number of sampling months = 3). Thus, a total of 90 samples (45 root and 45 soil samples) were used in this study, 30 and 15 root-soil sets at UTCBF and UTCF, respectively (Table 1).

### Soil physicochemical properties

Air-dried soil samples were sieved through a 500-µm mesh after litter removal and then 10 g was milled. Subsequently, 25–30-mg powdered soil samples were digested following a simplified perchloric acid digestion procedure (Kopacek and Hejzlar 2008). Total phosphorus (TP) was measured using BIOMOL Green Reagent (Enzo Life Science, NY, USA) following the manufacturer’s instructions. Total carbon (TC) and total nitrogen (TN) contents were analyzed by dry combustion of 25–30 mg of the powdered soil samples using an automatic highly sensitive NC analyzer SUMIGRAPH NC-22 F (Sumika Chemical Analysis Service, Ltd., Tokyo, Japan). The measured data were calibrated against an acetanilide standard. The ratio of TC to TN (C/N) also was calculated as one of the soil physicochemical properties. Soil pH was measured using a slightly modified procedure described in Djotan et al. (2022). Briefly, we added 50 mL of sterilized distilled water to 20 g of air-dried soil that had been passed through a 500-µm sieve and shook it for 5 min. Next, the mixtures were allowed to stand for 30 min and pH was measured using a compact pH meter (LAQUAtwin-pH-33; Horiba, Kyoto, Japan).

### DNA extraction and amplification

We used the sample processing procedures established by Djotan et al. (2022). Briefly, we removed root fragments and litter from freshly collected soil samples, washed the collected basal roots under running tap water, and selected all first- and second-order fine and fresh root fragments. The litter-removed soil sample and the selected root fragments were lyophilized and milled (separately). We extracted total DNA from 15 to 18 mg and 0.1 g of lyophilized root and soil samples, respectively, using the Extrap Soil DNA Kit Plus (ver. 2; BioDynamics Laboratory, Tokyo, Japan) according to the manufacturer’s instructions. Soil samples received 20 mg of skim milk each before DNA extraction. We amplified a ~550-bp fragment of the *rbc*L gene according to Djotan et al. (2022) to confirm the identity of the plant species of which the root samples were being investigated. For that, amplicons of the *rbc*L gene were sequenced with the Sanger method at Macrogen Japan (Tokyo, Japan).

For mycorrhizal fungi, we used two sets of primers, AML1/AML2 (Lee et al. 2008) followed by NS31/AM1 (Simon et al. 1992; Helgason et al. 1998), in a nested polymerase chain reaction (PCR) to amplify ~550 bp of the small subunit ribosomal DNA (SSU rDNA) and characterize the AMF communities in the roots and surrounding soils of *C. japonica*. In the first-round PCR, tenfold dilutions of the DNA aliquots were used as templates with the following cycling conditions: initial denaturation at 95 °C for 2 min; 30 cycles of 95 °C for 10 s, annealing at 58 °C for 45 s, and 72 °C for 60 s; and a final extension at 72 °C for 2 min. The products were diluted 100-fold and used as templates for the second-round PCR, where the same cycling conditions were used except the annealing temperature was set to 60 °C for 10 s. The Illumina adapters Tn5ME A and Tn5ME B were linked to the primers to allow sample multiplexing, as described in Djotan et al. (2022). The final PCR products were purified with AMPureXP beads (Beckman Coulter, CA, USA), multiplexed, and sent to Macrogen Japan for amplicon sequencing on the Illumina MiSeq platform (2 × 300 bp).

### Bioinformatics

To confirm that processed root samples originated from *C. japonica* trees and validate their inclusion in the AMF community investigation, the *rbc*L amplicon sequences were BLASTed against the NCBI GenBank database. Then, non-appropriate root samples and their corresponding soil samples were excluded from the AMF community investigation.

Bioinformatic analyses for the characterization of the AMF community were performed with QIIME 2 v. 2022.2.0 (Bolyen et al. 2019) unless otherwise stated. Paired-read sequences were processed using the pipeline and settings described in Djotan et al. (2023). In short, reads were demultiplexed, pairs were joined, and q-score filtering was applied. Then, sequences that passed the filters were used to define OTUs at 97% sequence similarity threshold. Chimeric sequences and rare OTUs (less than 10 reads across all samples or detected in only one sample) were discarded. Representative sequences of the OTUs were BLASTed against Maarj*AM* (Öpik et al. 2010), GlobalAMFungi (Větrovský et al. 2023), and National Center for Biotechnology Information (NCBI) GenBank databases using the NCBI-blast-2.10.0 + program (Morgulis et al. 2008) to discard non-Glomeromycotina OTUs. Taxa were assigned to OTUs based on the best matches to the abovementioned databases (query cover and percent identity > 95%), the consensus on AMF classification (Redecker et al. 2013) was used to update the taxonomic affiliation of the OTUs, and the composition of the AMF communities was visualized at the genus level. OTUs without good hit (query cover and percent identity < 95%) were not classified (labelled as “unclassified”), while OTUs that hit only “uncultured Glomeromycotina” in the databases were classified as “uncultured” and labelled as such.

The within-sample relative abundance of each OTU was computed, then averaged for each group of samples (6 sampling months × 2 compartments = 12 groups for UTCBF, 3 sampling months × 2 compartments = 6 groups for UTCF). Then, OTUs were ranked based on the decreasing order of their average relative abundance across all the 18 groups of samples (both sites). An OTU was categorized as dominant when its average relative abundance at both study sites was higher than or equal to 1%. The dominant OTUs were further categorized as persistent (P, detected in all sampling months) and seasonal (S, when not detected in one or more sampling months). Sequences of the dominant OTUs were aligned using MEGA11 and positioned on a phylogenetic tree. The tree was annotated and displayed using FigTree v.1.4.4 (http://tree.bio.ed.ac.uk/software/figtree/).

### Statistical analysis

Statistical analyses were performed with R v.4.3.2 (R Core Team 2023). We estimated alpha diversity indices with vegan R package v. 2.6-4 and tested their variation between compartments (root vs. soil) and between sampling months (May, July, September, November, January, and March) using ANOVA followed by Tukey’s honestly significant difference (HSD) test at a 95% confidence level when a factor or the interaction of factors exerted significant effects on the alpha diversity. Shapiro-Wilk normality test and Levene’s test for homogeneity of variance were performed to confirm normality and equality of variance prior to parametric tests. We tested the variation in the soil physicochemical properties between sites and sampling months and compared the mean values using the same previously described tests.

Using permutational analysis of variance (PERMANOVA) with *adonis2* in the vegan R package, we tested the variation in the AMF communities between sites, compartments, and sampling months. Within each site, variations in root and soil AMF communities of *C. japonica* across sampling months were tested with PERMANOVA. Then, *pairwise.adonis* for multilevel pairwise comparison using adonis2 from the vegan R package was employed to identify months with similar and dissimilar AMF communities in the roots and in the soils. The AMF community at each site was ordinated and visualized using the ggplot2 R package v. 3.4.0. We analyzed the relationships between soil physicochemical properties and AMF communities in the roots and surrounding soils of *C. japonica* by Mantel test and redundancy analysis (RDA) in the vegan R package. In the Mantel test, we used Euclidean distance for pH, TC, TN, C/N, and TP, and Bray-Curtis’ distance for the AMF community matrix to calculate the Spearman correlations. The RDA model was tested with PERMANOVA.

## Results

### Soil physicochemical properties

Except for pH, all soil physicochemical properties (TC, TN, C/N, and TP) significantly differed between sites (Table S2). At UTCBF, pH and TP significantly differed among sampling months but only TP differed significantly at UTCF (Tables 1 and S2). At UTCBF, lower pH values were observed in September while higher values were observed in March. TP decreased from May to September, and then increased in November before decreasing again sharply in January through March. TC, TN, and C/N were in the ranges of 6.29–10.29%, 0.45–0.74%, and 13.00–16.42, respectively. At UTCF, TP was higher in May than in November and March. pH, TC, TN, and C/N were in the ranges of 4.78–5.29, 16.48–17.70%, 1.01–1.06%, and 15.79–16.64, respectively.

### Sequencing summary

From 45 confirmed *C. japonica* root samples and 45 corresponding soil samples, a total of 1,149,207 amplicon sequences were obtained, which clustered into 7625 OTUs after trimming, pair-joining, and denoising. After removing chimeric, non-Glomeromycotina, and rare amplicon sequences, 962,861 sequences remained, which clustered into 698 OTUs. The number of AMF amplicon sequences per sample ranged from 3537 to 16,435. The sequence read archives were deposited in the National Center for Biotechnology Information database (PRJNA898865); the representative nucleotide sequences of the AMF OTUs (OR577640–OR578337) and the representative partial nucleotide sequence of *rbc*L for *C. japonica* (OP832016) were deposited in GenBank SUB13854369 and BankIt2642437, respectively.

### Composition of AMF communities in roots and soils of *C. japonica* across sampling months

**Fig. 1:**
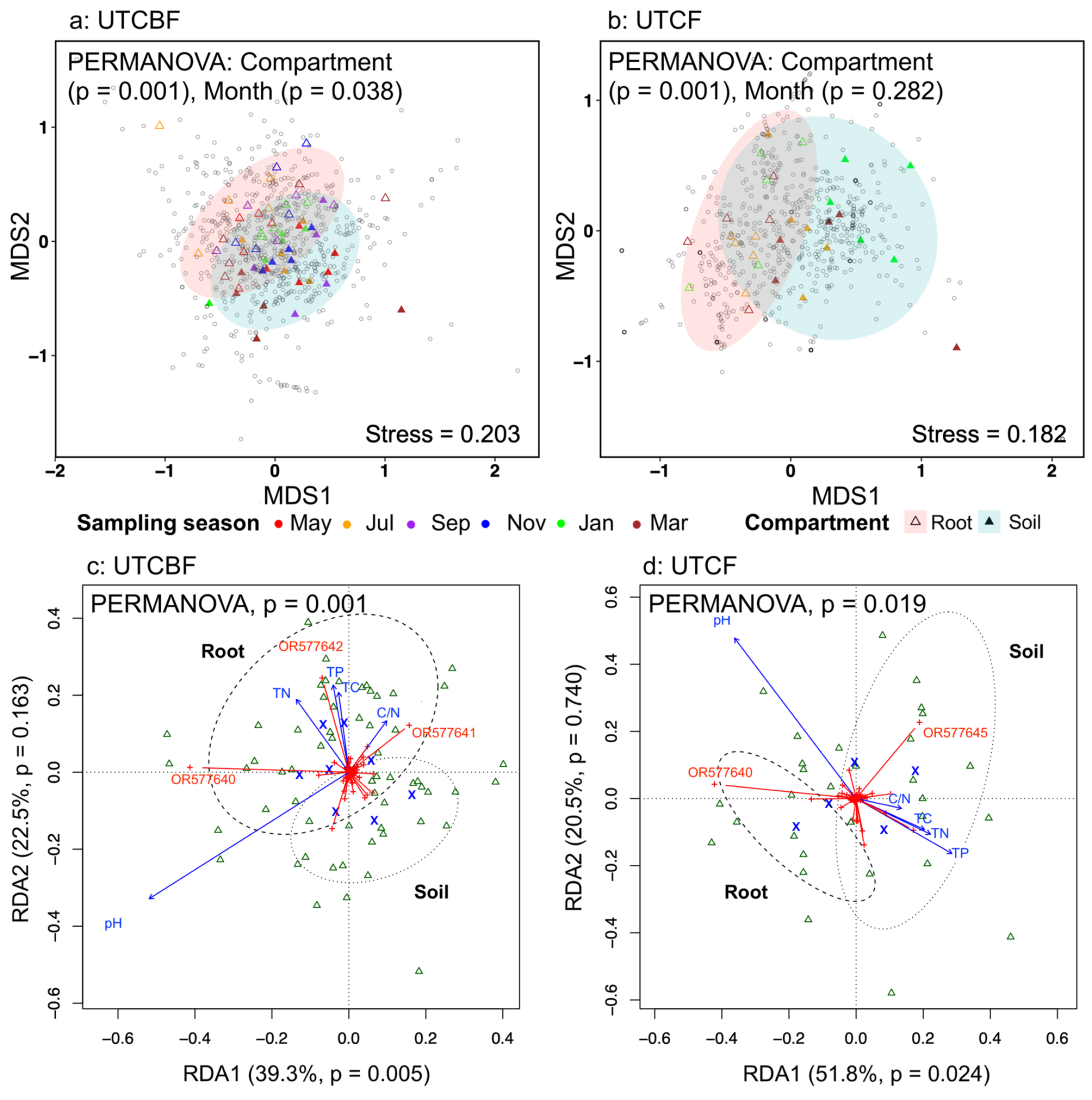
Non-metric multidimensional scaling (NMDS, **a** and **b**) and triplots of redundancy analysis (RDA, **c** and **d**) of the arbuscular mycorrhizal fungi (AMF) in roots and soils of *Cryptomeria japonica* investigated seasonally at the University of Tokyo Chiba (UTCBF, **a** and **c**) and Chichibu (UTCF, **b** and **d**) Forests.  NMDS followed by a permutational analysis of variance (PERMANOVA, **a**, **b**) revealed significant differences between root and soil AMF communities in *C. japonica* at each site, and significant seasonality (variation between sampling months) at UTCBF but not at UTCF. For each site, permutation tests of RDA revealed significant linear relationships between relative abundance of OTUs and soil pH but not with other physicochemical properties (**c**, **d**). The most influential OTUs at each site are labeled with their accession numbers in red on the triplots. TC, total carbon; TN, total nitrogen; C/N, carbon to nitrogen ratio; TP; total phosphorus; △, samples; ×, positions for levels of factors (site, sampling month, and compartment)

**Fig. 2:**
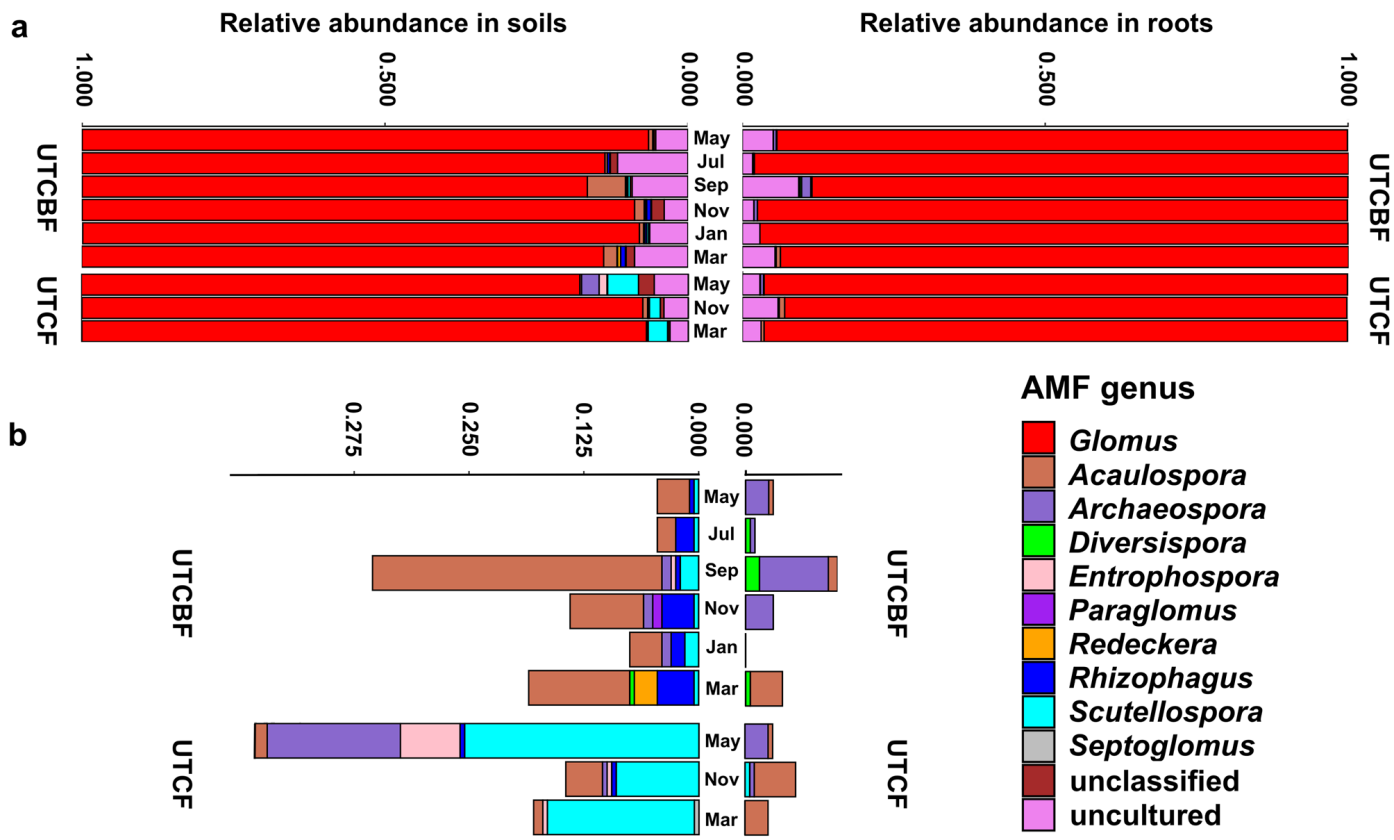
Taxonomic composition of the AMF communities in roots and surrounding soils of *Cryptomeria japonica*, showing all AMF OTUs (**a**) and excluding AMF OTUs assigned to *Glomus* spp., and those categorized as “unclassified” and “uncultured” (**b**) in each sampling month.  This community composition was based on BLAST searches. OTUs without good hit (query cover and percent identity < 95%) where not classified (labelled as “unclassified”) while OTUs that hit only “uncultured Glomeromycotina” in the databases were classified as “uncultured” and labelled as such

The OTU richness of the *C. japonica* AMF community did not significantly differ between sites (UTCBF and UTCF), compartments (root and soil), and sampling months (May, July, September, November, January, and March), but the Shannon diversity significantly differed between root and soil (Tables 1 and S3, Online Resources 3 and 4). The composition of the AMF communities at each site significantly differed between compartments but the community within each compartment did not vary significantly between sampling months (Fig. 1, Table S4). However, the total AMF community (root + soil) at UTCBF but not UTCF showed significant seasonal variation due to different assemblages in May, July, and September (Table S5). Among the 698 AMF OTUs recovered from the roots and surrounding soils of *C. japonica* collected at UTCBF and UTCF, 575 belonged to Glomeraceae, dominated by *Glomus* (Fig. 2, Table S6). In the decreasing order of the number of OTUs, Acaulosporaceae, Diversisporaceae, Archaeosporaceae, Gigasporaceae, Entrophosporaceae, and Paraglomeraceae also were detected. In addition, 75 OTUs were classified as “uncultured,” and 26 were not classified. At UTCBF, 156 of 604 and 165 of 618 OTUs were detected in all six sampling months in the roots and soils, respectively (Online Resource 5). At UTCF, 225 of 527 and 232 of 538 OTUs were detected in all three sampling months in the roots and soils, respectively. The relative abundance of *Glomus* was highest in both compartments (roots and soils) in all sampling months regardless of site, while other genera such as *Archaeospora*, *Acaulospora*, *Rhizophagus*, and *Scutellospora* showed seasonal shifts between roots and soils over sampling months (Fig. 2).

**Table 1:** Soil physicochemical properties and alpha diversity of root and surrounding soil AMF communities of *Cryptomeria japonica* investigated seasonally at UTCBF and UTCF over a year

Sampling date	Soil physicochemical properties					Number of OTUs		Shannon index	
	pH	TC (%)	TN (%)	C/N	TP (mg. kg^−1^. soil)	Root	Soil	Root	Soil
A: University of Tokyo Chiba Forest (UTCBF)									
May 26	4.74 ± 0.47 ab	10.29 ± 4.84 a	0.65 ± 0.26 a	15.42 ± 2.11 a	81.43 ± 25.03 ab	176 ± 42 a	155 ± 22 a	2.55 ± 0.32 a	2.50 ± 0.24 a
July 21	5.02 ± 0.24 ab	10.07 ± 1.95 a	0.61 ± 0.10 a	16.42 ± 1.82 a	76.33 ± 18.03 ab	167 ± 38 a	163 ± 14 a	2.36 ± 0.76 a	2.77 ± 0.21 a
September 24	4.61 ± 0.45 b	6.29 ± 2.57 a	0.45 ± 0.15 a	13.78 ± 1.44 a	59.30 ± 18.65 b	136 ± 21 a	144 ± 32 a	2.43 ± 0.22 a	2.70 ± 0.34 a
November 24	5.23 ± 0.39 ab	8.44 ± 3.65 a	0.55 ± 0.19 a	14.91 ± 1.74 a	89.58 ± 18.42 a	139 ± 20 a	150 ± 24 a	2.40 ± 0.23 a	2.83 ± 0.08 a
January 31	5.10 ± 0.53 ab	9.45 ± 3.67 a	0.74 ± 0.29 a	13.00 ± 3.06 a	31.33 ± 9.40 c	150 ± 35 a	155 ± 33 a	2.57 ± 0.39 a	2.81 ± 0.18 a
March 23	5.43 ± 0.37 a	7.14 ± 2.85 a	0.48 ± 0.16 a	14.70 ± 1.52 a	28.86 ± 8.54 c	144 ± 25 a	161 ± 26 a	2.59 ± 0.20 a	2.90 ± 0.29 a

B: University of Tokyo Chichibu Forest (UTCF)									
May 19	4.78 ± 0.33 a	16.84 ± 3.41 a	1.06 ± 0.18 a	15.79 ± 0.71 a	81.19 ± 9.85 a	138 ± 21 a	145 ± 22 a	2.42 ± 0.49 a	2.87 ± 0.23 a
November 17	5.29 ± 0.40 a	17.70 ± 3.18 a	1.06 ± 0.15 a	16.64 ± 0.84 a	69.19 ± 5.15 b	156 ± 11 a	162 ± 20 a	2.77 ± 0.28 a	2.95 ± 0.26 a
March 22	5.09 ± 0.23 a	16.48 ± 2.01 a	1.01 ± 0.11 a	16.29 ± 0.31 a	70.62 ± 6.62 b	140 ± 26 a	133 ± 36 a	2.53 ± 0.47 a	2.45 ± 0.83 a

Values are presented as the average ± standard deviation (*n* = 5) for soil pH, total carbon (TC), total nitrogen (TN), ratio of TC to TN (C/N), and total phosphorus (TP)

Within the same site and for the same variable (in column), groups with the same letter are not significantly different by Tukey HSD test following one-way ANOVA. All soil physicochemical properties except pH were significantly different between sites (Table S2)

The number of OTUs did not significantly differ between root and soil compartments but Shannon index did differ (Table S3)

### Dominant AMF OTUs in the roots and surrounding soils of *C. japonica* across sampling months

Overall, we detected 24 dominant OTUs, 23 of which were continuously detected in the roots and surrounding soils while one was seasonal in the roots but continuously detected in the soils (OR577659) (Tables 2 and S7, Online Resource 6). The cumulative proportion of dominant OTUs at a sampling ranged from 72.9 to 91.2%, while the average proportion of a single dominant OTU ranged from 1.0 to 18.4% across sampling months (Tables 2 and S7). Phylogenetically, two of the dominant OTUs were placed in *Glomus* (OR577645 and OR577659); the OTUs OR577647, OR577663, OR577654, OR577642, and OR577653 were placed in *Diversispora*, *Dominikia*, *Microkamienskia*, *Rhizophagus*, and *Septoglomus*, respectively; and 17 OTUs including the top two (OR577641 and OR577640) were positioned in different unknown genus-level clades in Glomeraceae (Online Resource 7, Table 2). Based on their placement, the top two dominant OTUs OR577640 and OR577641 are potentially *Dominikia* and *Rhizophagus* AMF, with overall average relative abundances across all sampling months and sites of 17.4% and 18.4%, respectively. These two OTUs were detected in the roots of all *C. japonica* trees in all sampling months and sites (Table S7). Their relative abundances showed seasonality at both sites but with different variation patterns (Online Resource 8).

### Relationships between soil physicochemical properties and AMF communities in the roots and surrounding soils of *C. japonica* across sampling months

Based on the permutation test, the RDA models were significant for the AMF communities at both sites (Fig. 1). At UTCBF (UTCF), the first two axes explained 61.8% (72.2%) of the total variance, the first axis alone explaining 39.3% (51.8%), but only the first axis showed significant linear relationships between the composition of the AMF community and the soil physicochemical properties. The variations observed in the *C. japonica* AMF community were significantly explained by sample type (factor: compartment, levels: root and soil) but not by sampling month (Table S8). We detected 87 and 36 OTUs that showed significant correlation with environmental conditions including soil properties at UTCBF and UTCF, respectively. Of them, four (OTUs OR577640, OR577641, OR577642, and OR577645) had correlation coefficients higher or equal to 0.5 and *p*-values = 0.001, and on the RDA plots were shown to be very influential OTUs in the community (Fig. 1, Tables S8 and S9). Soil pH was significantly correlated with OTUs OR577640, OR577641, and OR577642 at UTCBF and OTUs OR577640 and OR577645 at UTCF (Fig. 1). Mantel test results showed that only the covariation between soil AMF community and soil pH was significant (*r* = 0.146, *p* = 0.04) at UTCBF (Table S10). At UTCF, only soil pH showed significant covariations with the root AMF community (*r* = 0.449, *p* = 0.00) and the total AMF community (*r* = 0.198, *p* = 0.02).

**Table 2:** Dominant operational taxonomic units (OTUs) of AMF in the roots and surrounding soils of *Cryptomeria japonica* investigated seasonally at UTCBF and UTCF

Accession of dominant OTUs^a^	BLAST placement of the OTUs based on MaarjAM, NCBI, and GlobalAMFungi databases^b^				Phylogenetic placement of the OTUs (Genus level)^c^	Yearlong average abundance at both sites
	Match’s accession	Query cover	Per. Id	Genus or description		
OR577641	VTX00080	99.62	99.62	*Glomus*	unknown	0.184
OR577640	VTX00166	99.81	99.81	*Glomus*	unknown	0.177
OR577642	VTX00113	99.81	99.81	*Glomus*	*Rhizophagus*	0.046
OR577643	VTX00224	99.81	99.81	*Glomus*	unknown	0.042
OR577644	VTX00084	97.50	97.50	*Glomus*	unknown	0.038
OR577646	VTX00219	99.23	99.23	*Glomus*	unknown	0.033
OR577645	VTX00199	99.81	99.81	*Glomus*	*Glomus*	0.023
OR577647	AJ563878	98.53	98.53	uncultured	*Diversispora*	0.032
OR577648	VTX00088	98.08	98.08	*Glomus*	unknown	0.028
OR577649	VTX00291	98.46	98.46	*Glomus*	unknown	0.027
OR577650	VTX00088	98.27	98.27	*Glomus*	unknown	0.027
OR577651	VTX00084	97.34	97.34	*Glomus*	unknown	0.025
OR577652	VTX00128	98.08	98.08	*Glomus*	unknown	0.018
OR577653	VTX00117	99.42	99.42	*Glomus*	*Septoglomus*	0.017
OR577654	VTX00122	99.81	99.81	*Glomus*	*Microkamienskia*	0.017
OR577655	VTX00291	97.92	97.92	*Glomus*	unknown	0.015
OR577657	VTX00126	98.85	98.85	*Glomus*	unknown	0.011
OR577656	VTX00088	97.50	97.50	*Glomus*	unknown	0.014
OR577658	VTX00219	97.89	97.89	*Glomus*	unknown	0.012
OR577659	VTX00103	99.81	99.81	*Glomus*	*Glomus*	0.014
OR577662	VTX00080	96.54	96.54	*Glomus*	unknown	0.011
OR577663	VTX00125	99.81	99.81	*Glomus*	*Dominikia*	0.009
OR577661	VTX00083	99.04	99.04	*Glomus*	unknown	0.010
OR577660	VTX00072	99.62	99.62	*Glomus*	unknown	0.010

^a^The within-sample relative abundance of each OTU was computed, then averaged for each group of samples (6 sampling months × 2 compartments = 12 groups for UTCBF, 3 sampling months × 2 compartments = 6 groups for UTCF). The table is sorted by the average relative abundance of the dominant OTUs across sampling months at both sites (> 0.01)

^b^Taxa were assigned to OTUs only when both query cover and percent identity were ≥ 95%; Per. Id, percent identity to the closest match; the description “uncultured” refers to OTUs for which the best matches were described as such in the databases

^c^Results of maximum likelihood phylogenetic placement of OTUs. “unknown” indicates unknown position in Glomeraceae. Except for OTU OR577659 which was seasonal in roots and permanent in soils, all other dominant OTUs were permanently detected in both compartments

## Discussion

### Dynamics of soil physicochemical properties

Soil pH and TP showed significant seasonal variations, while TC, TN, and C/N did not. Meanwhile, only pH showed significant correlation and covariation with the AMF community. At UTCBF, soil pH was lower at the end of September but higher at the end of March; this pattern of variation may be related to rainfall in summer (increasing pH) and the microbial activity triggered by the start of the growing season (decreasing pH). Soil TP increased from March to July, decreased from July to September, and then increased again in November before falling again in January. This variation pattern indicates that TP fluxes between soil and *C. japonica* plants are higher in the growth season than in winter but may encounter interference due to heavy rains.

### Seasonal dynamics of AMF communities

In this study, AMF communities were significantly different between roots and surrounding soils of *C. japonica* in every sampling month but the two habitats had similar OTU richness year-round. This result suggests that variation in the number of AMF species in roots vs. surrounding soils is a result of stochastic processes, while the species composition is a result of deterministic processes driven by host selectivity. Therefore, a comparison of OTU richness between roots and soils alone (alpha diversity only) cannot reveal the AMF community dynamics in the two habitats. Our findings support a report that both stochastic and deterministic processes determine the dynamics of the AMF community (Dumbrell et al. 2010). Previous studies of AMF communities in the two habitats can be divided into two groups based on their findings regarding OTU richness in roots vs. soils: in one, the roots had higher OTU richness than the soil (Wang et al. 2015; Berruti et al. 2017; Mahmoudi et al. 2019; Djotan et al. 2023), while in the other, the soil had higher OTU richness than the roots (Xu et al. 2017; Faggioli et al. 2019; Gu et al. 2022; Djotan et al. 2022; Faghihinia et al. 2022). These studies were carried out in different systems and cannot reveal the dynamics of OTU richness in or between the roots and soils of host plants. However, in our current seasonal study of the AMF assemblages of *C. japonica*, which was carried out simultaneously on the roots and surrounding soils of the same host species in the same environments, and with the same methodology, we clarified that root and soil AMF communities have the same species richness but the constituent species differ between the two habitats. Additionally, applying the same DNA extraction kit to the two different sample types (root and soil) might have substantially clarified AMF diversity in the roots and surrounding soils of the same host plants.

The composition of the total AMF community (roots + soil) showed significant seasonality at UTCBF but not at UTCF where only data from three sampling months (May, November, and March) were used. At UTCBF, the total AMF assemblage in May was significantly different from those in July and September (multiple pairwise PERMANOVA, *p* < 0.05), explaining the seasonal variation observed at the site. Thus, it is likely that significant seasonal variation was not detected at UTCF because AMF communities in July and September were not included in the analysis. Although pH and TP showed significant seasonal variations, their values did not differ significantly from May to September at UTCBF (but a decrease was observed in TP from May to September), suggesting that the variation observed in the root AMF communities between May and July at the site could be attributed to random effect or to other factors such as precipitation, air temperature, and solar radiation, which had increased significantly between the two sampling months, potentially affecting the soil conditions, plant physiology, and microbial activities (Table S1).

When seasonal variation was tested separately for root and soil AMF communities at each site, we found that neither the root nor soil AMF community showed significant seasonal variation although the total AMF community has shown significant seasonality at UTCBF. These results serve as a warning that the dual-habitat lifestyle of AMF should be considered in the study of their community ecology. Our finding regarding the seasonality of the root AMF community of *C. japonica* trees in forest ecosystems opposes that by Dumbrell et al. (2011), who found that root AMF communities investigated in a grassland ecosystem differed between summer and winter. These findings in a forest vs. grassland ecosystem suggest that AMF assemblages in the roots of short-lived plants may be more prone to seasonal changes than those in the roots of long-lived plants. However, our finding regarding the seasonality of the soil AMF community of *C. japonica* trees in forest ecosystems supports a report that AMF communities in forest soil do not vary throughout the growing season (Davison et al. 2012). At both sites, > 25% of the OTUs were detected in all sampling months and very few OTUs were temporal in the roots and surrounding soils of *C. japonica*. The relative abundance of seasonally detected OTUs was lower than that of the persistently dominant OTUs. This composition of the AMF community at the OTU level may explain the weak seasonality observed in the current study. These findings also evoke the theoretical driver/passenger hypothesis in successional AMF community dynamics proposed by Hart et al. (2001). In established vegetation where plant community dynamics are minimal (almost no change in the plant community), the AMF community might also remain stable.

### Soil pH determines the composition and dynamics of the AMF community associated with *C. japonica*

Soil pH but not TC, TN, C/N, and TP was an excellent linear predictor of the composition of the AMF community associated with *C. japonica*. Among the 116 OTUs (from both sites) showing significant correlations with environmental factors including soil properties, four (the OTUs OR577640, OR577641, OR577642, and OR577645) showed very strong and significant correlations with soil pH but differently at the different sites. OTUs OR577640, OR577641, and OR577642 showed significant correlations with soil pH at UTCBF, while OR577640 and OR577645 were correlated with pH at UTCF. A molecular survey of AMF from 425 individual plants of 28 species along a soil pH gradient similarly found that although the AMF community is influenced by stochastic processes, it still responds predictably to soil pH (Dumbrell et al. 2010). Our findings corroborate the significant effects of soil pH on the AMF community. However, it contradicts reports that soil carbon and nitrogen strongly influence the composition of AMF communities (Qiang et al. 2019; Guan et al. 2020). OTUs OR577640 and OR577641 belong to different genus-level clades in Glomeraceae and exhibited different relationship with pH. This finding supports the report that relationships between individual AMF taxa and soil pH may be phylogenetically conserved at the genus level within the Glomeromycotina (Bainard et al. 2014).

Partitioning the AMF community into core and satellite communities could increase the variation explained by microbial community analyses (Barnes et al. 2016). Although this might clarify the stochasticity associated with the satellite community and the determinism associated with the core community, doing so would create two artificial community datasets, and analyses may miss the determinant ecological aspects of the AMF community. Thus, we suggest that the responses of dominant OTUs in a community to environmental factors should be investigated to understand the effects of those factors on that community.

### Biogeography and ecology of the dominant AMF associated with *C. japonica*

We detected 24 dominant OTUs based on our proposed method. Among them, two OTUs (OR577641, likely *Rhizophagus* and OR577640, likely *Dominikia*) showed absolute dominance in the community, with average relative abundances > 10%, reflecting the definition of “co-dominant” by Kikvidze and Ohsawa (2002). However, when the method of Kikvidze and Ohsawa (2002) was applied, the community appeared to have 13 dominant OTUs (the top 13 in the list of 24 OTUs). Thus, compared to their method, our method increased the number of apparent dominant OTUs in the community. The top two AMF OTUs that persisted regardless of the year-round changes in the environment across sites were OR577641 (VTX00080) and OR577640 (VTX00166) with average relative abundances of 18.4% and 17.4%, respectively. The OTU OR577640 was more abundant in roots than in soils year-round, while the OTU OR577641 was more abundant in soils than in roots in May, July, and January, but more abundant in roots than in soils in September, November, and March. Their relative abundances increased in the roots in September and November, which correspond to the period when fine root production in *C. japonica* was highest according to Konôpka et al. (2006). We detected them in all investigated individual trees of *C. japonica* in all sampling months and at all sites. Their relative abundances were negatively correlated, suggesting an antagonistic relationship and competition for habitat occupancy. These two dominant OTUs are globally distributed in forest and grassland ecosystems, respectively (Větrovský et al. 2023). According to the database, VTX00080 is equally distributed in roots (44.56%) and soils (44.3%), and more in forest (76.39%) than in cropland (18.44%) ecosystems; VTX00166 is more distributed in roots (66.25%) than in soils (28.84%), and equally distributed in grassland (39.27%) and forest (40.17%) ecosystems. Our results coincided with the information retrieved from the database and added root-soil seasonality information to understanding the ecology and biogeography of these two globally distributed OTUs. The dominant OTUs OR577641 and OR577640 were also detected and declared dominant in other *C. japonica* plantations (Djotan et al. 2022, 2023).

In the study by Djotan et al. (2023), one OTU (MZ479751, which was described as a *Paraglomus* but is likely to be an unknow AMF genus in Glomeraceae) was particularly more abundant in surrounding soil samples than in roots of *C. japonica*, constituting a large proportion of the AMF community detected in the soil. However, we did not detect similar OTUs in the current study. It is well established that geographical distance and the local environment, including soil type, pH, and carbon content, determine the AMF community composition at fine spatial scales (Hazard et al. 2013; Bouffaud et al. 2016; Nacke et al. 2016; Goldmann et al. 2020), explaining the contrasting results between studies. On the other hand, the detection of AMF taxa in the roots of a host may depend on its colonization levels by the AMF which could be below the limit of molecular detection. Also, the DNA extraction method (such as the kit) and the targeted DNA barcode (determined by primer sets) could also contribute to the non-detection of some AMF taxa in roots and soils. All the dominant OTUs except one were assigned to *Glomus* spp. based on BLAST search, but the phylogenetic analysis indicated that some of them belong to unknown genus-level clades, *Dominikia*, *Diversispora*, *Microkamienskia*, and *Rhizophagus*. Thus, based on the above, we suggest that a phylogenetic analysis of all detected AMF OTUs would provide an accurate composition of the AMF community at the genus level.

In this study, we hypothesized that there are significant shifts in and between root and soil AMF communities of *C. japonica* across seasons under the influence of variations in soil physicochemical properties. This hypothesis was partly confirmed. AMF communities in roots and soils of *C. japonica* are different in all seasons but without significant seasonality. However, the total AMF community (roots + soil) changes significantly as we move from May to September. Year-round, the richness of AMF species in the roots and surrounding soils of *C. japonica* remains the same, but the specific AMF assemblages differ between the two habitats. Thus, with their lifestyle, AMF may have high environmental plasticity to sustain a functional symbiosis in constantly changing environments.

### Electronic supplementary material

Below is the link to the electronic supplementary material.

Supplementary Material 1 (DOCX 3490 KB)

Supplementary Material 2 (XLSX 42.6 KB)

## Data Availability

The sequence read archives were deposited in the National Center for Biotechnology Information database (PRJNA898865), the representative nucleotide sequences of the AMF OTUs (OR577640–OR578337) and and the representative partial nucleotide sequence of *rbc*L for *C. japonica* (OP832016) were deposited in GenBank SUB13854369 and BankIt 2642437, respectively.
