# Supplementary material for: Year-round dynamics of arbuscular mycorrhizal fungi communities in the roots and surrounding soils of Cryptomeria japonica
Source: Mycorrhiza. 2024 Mar 20;34(1-2):119–30. doi: 10.1007/s00572-024-01143-x (PMC10998819; doi:10.1007/s00572-024-01143-x)
Supplement: Supplementary file 1 — Supplementary Material 1 (DOCX 3490 KB) [file 572_2024_1143_MOESM1_ESM.docx]

**Year-round dynamics of arbuscular mycorrhizal fungi community in the roots and surrounding soils of *Cryptomeria japonica***

Akotchiffor Kevin Geoffroy Djotan ^*1, 2^, Norihisa Matsushita^1, 3^, Kenji Fukuda^1, 4^

**^1^**University of Tokyo, Graduate School of Agricultural and Life Sciences (Laboratory of Forest Botany) 1-1-1, Yayoi, Bunkyo, Tokyo, 113-8657, Japan

ORCID: ^2^https://orcid.org/0000-0002-3726-9826; ^3^https://orcid.org/0000-0003-3281-8846, ^4^https://orcid.org/0000-0002-9980-3107

*^*^Corresponding author, E-mail: geoffroydjotan@yahoo.fr*


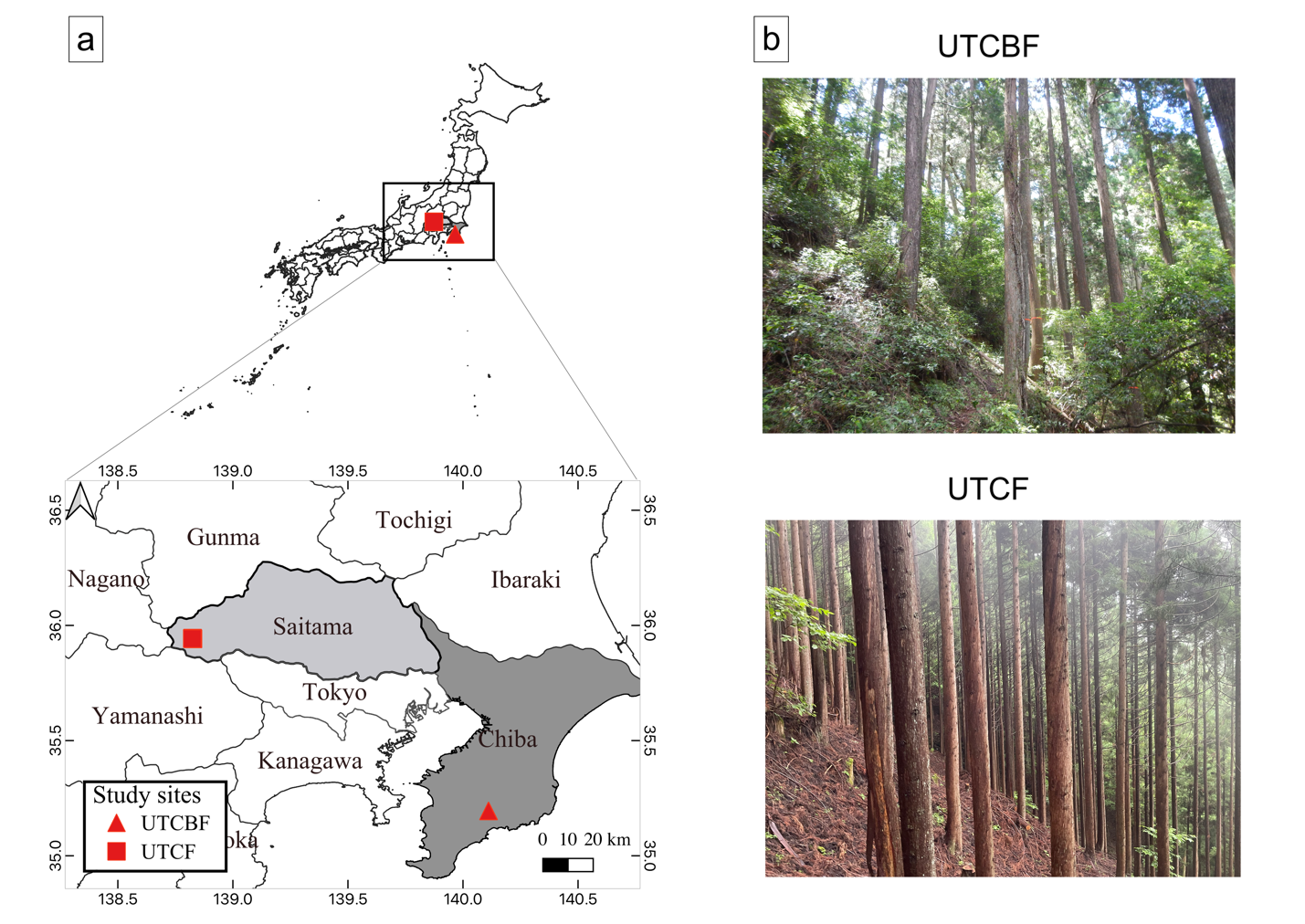


Online Resource 1 Locations of the study sites (a) and physiognomy of the vegetation below the canopy layer (b)

UTCBF, University of Tokyo Chiba Forest; UTCF, University of Tokyo Chichibu Forest
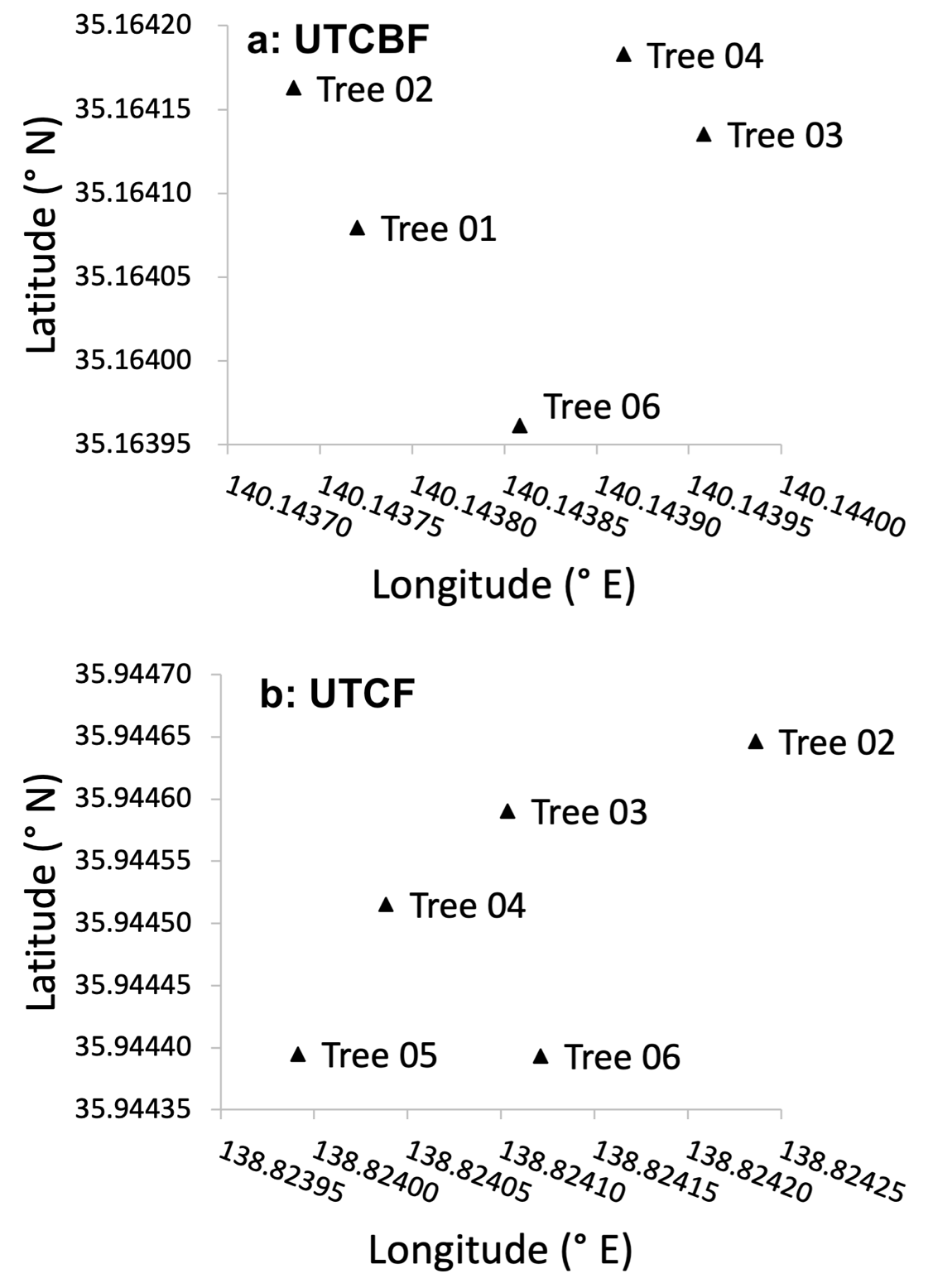


Online Resource 2 Spatial distribution of the seasonally investigated trees of Cryptomeria japonica at the University of Tokyo Chiba (a) and Chichibu (b) forests in central Japan

Labels of the trees at the two sites as shown on the figure are independent.


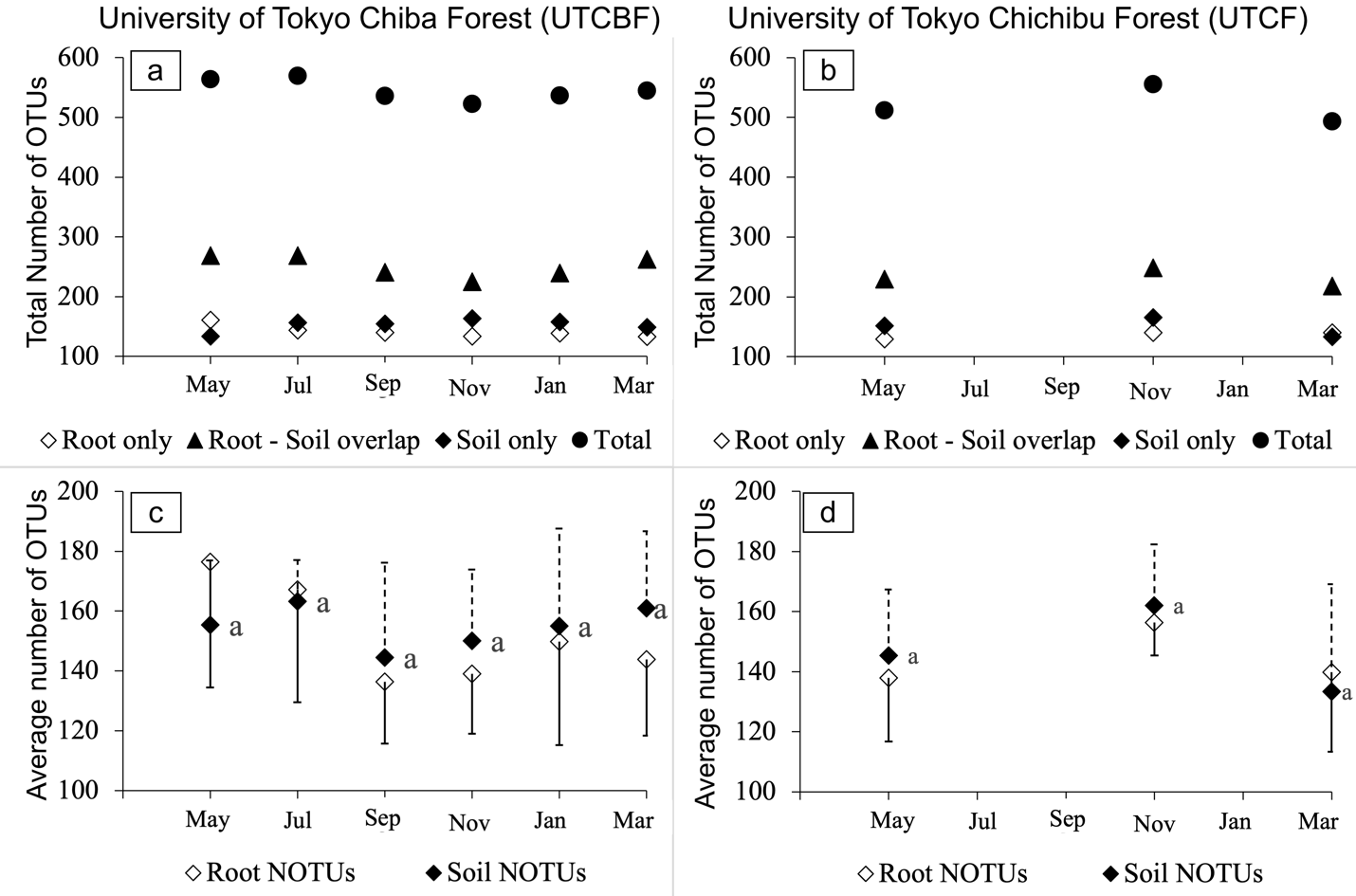


Online Resource 3 Dynamics of total OTU richness (a, b) and average OTU richness (c, d) in root and soil AMF communities of Cryptomeria japonica investigated seasonally at two sites

AMF, arbuscular mycorrhizal fungi; OTUs, operational taxonomic units; UTCBF, University of Tokyo Chiba Forest; UTCF, University of Tokyo Chichibu Forest. The full and dashed vertical lines on panel c and d show the standard deviation for root and soil OTU richness, respectively.


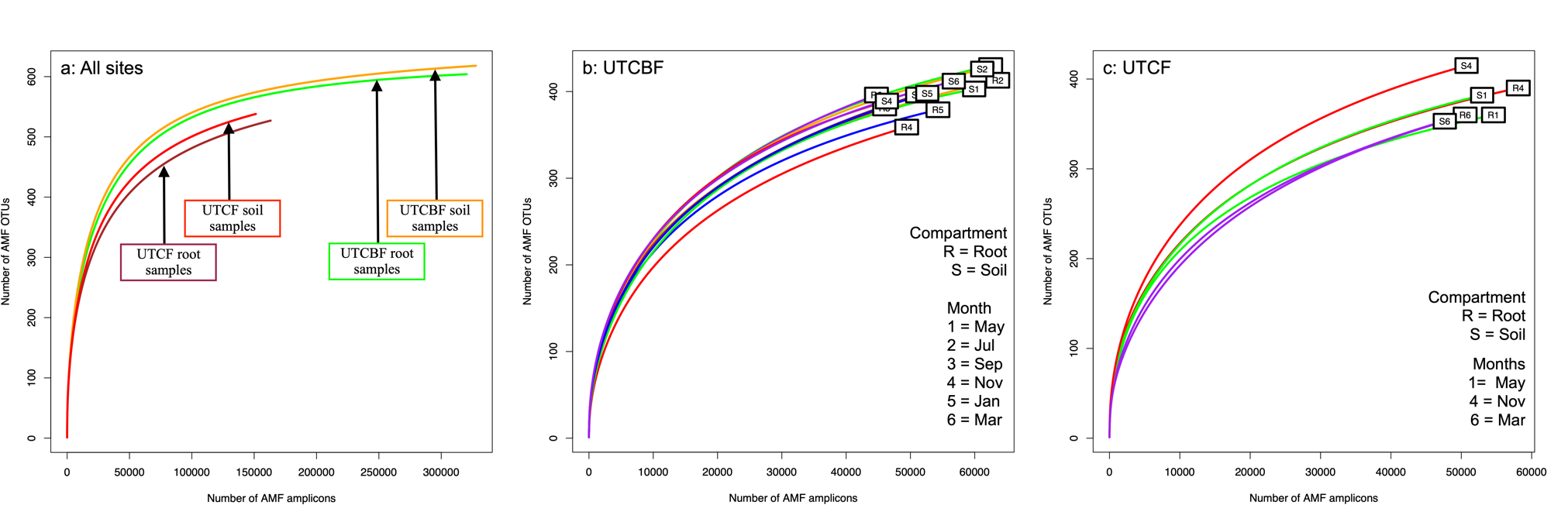


Online Resource 4 Accumulation curves of AMF OTUs detected at all sites (a), UTCBF (b), and UTCF (c)

AMF, arbuscular mycorrhizal fungi; OTUs, operational taxonomic units; UTCBF, University of Tokyo Chiba Forest; UTCF, University of Tokyo Chichibu Forest.


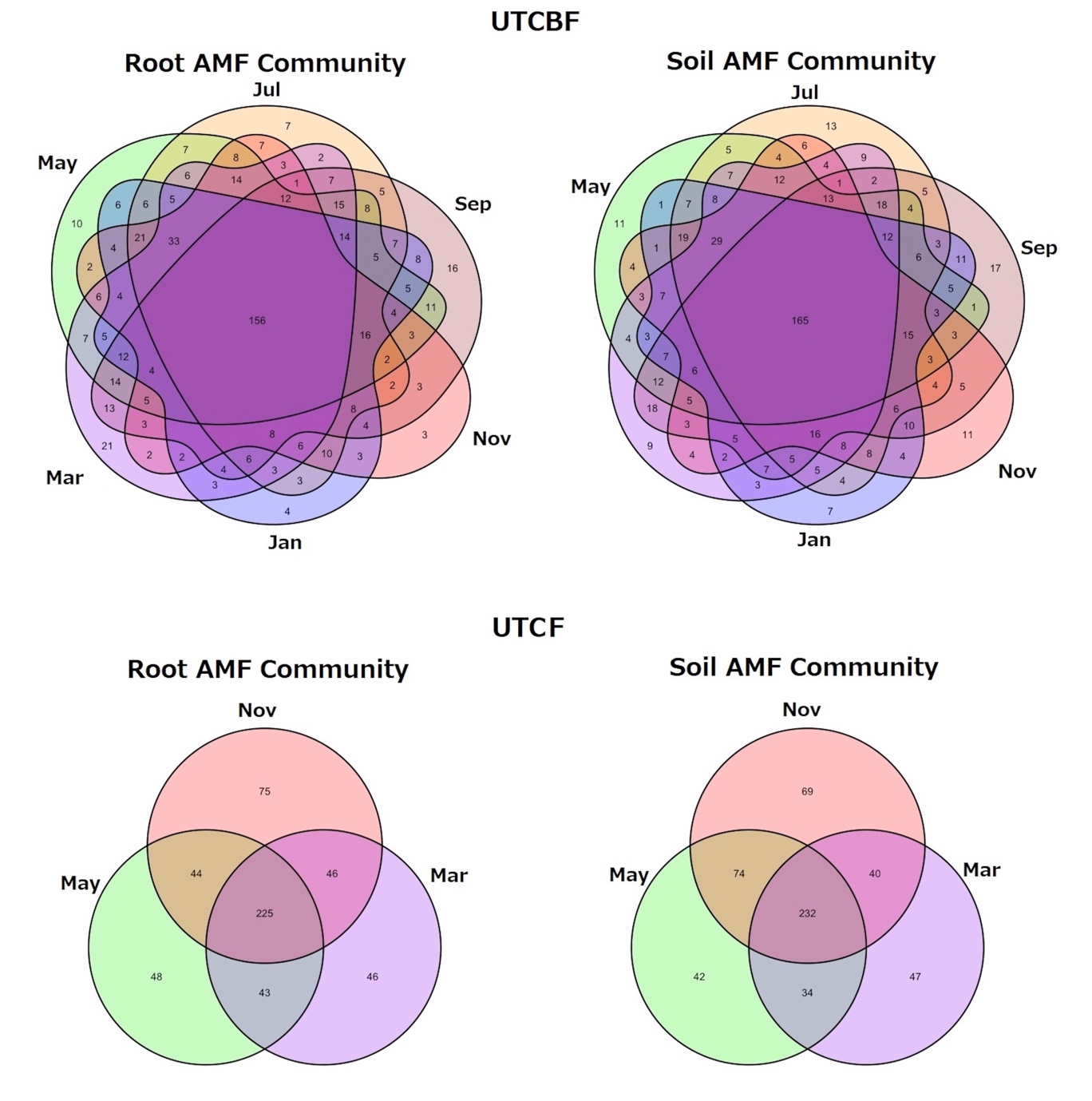


Online Resource 5 Venn diagram showing the numbers of persistent and seasonal AMF OTUs recovered from root and soil samples of Cryptomeria japonica investigated at two sites

AMF, arbuscular mycorrhizal fungi; OTUs, operational taxonomic units; UTCBF, University of Tokyo Chiba Forest; UTCF, University of Tokyo Chichibu Forest.


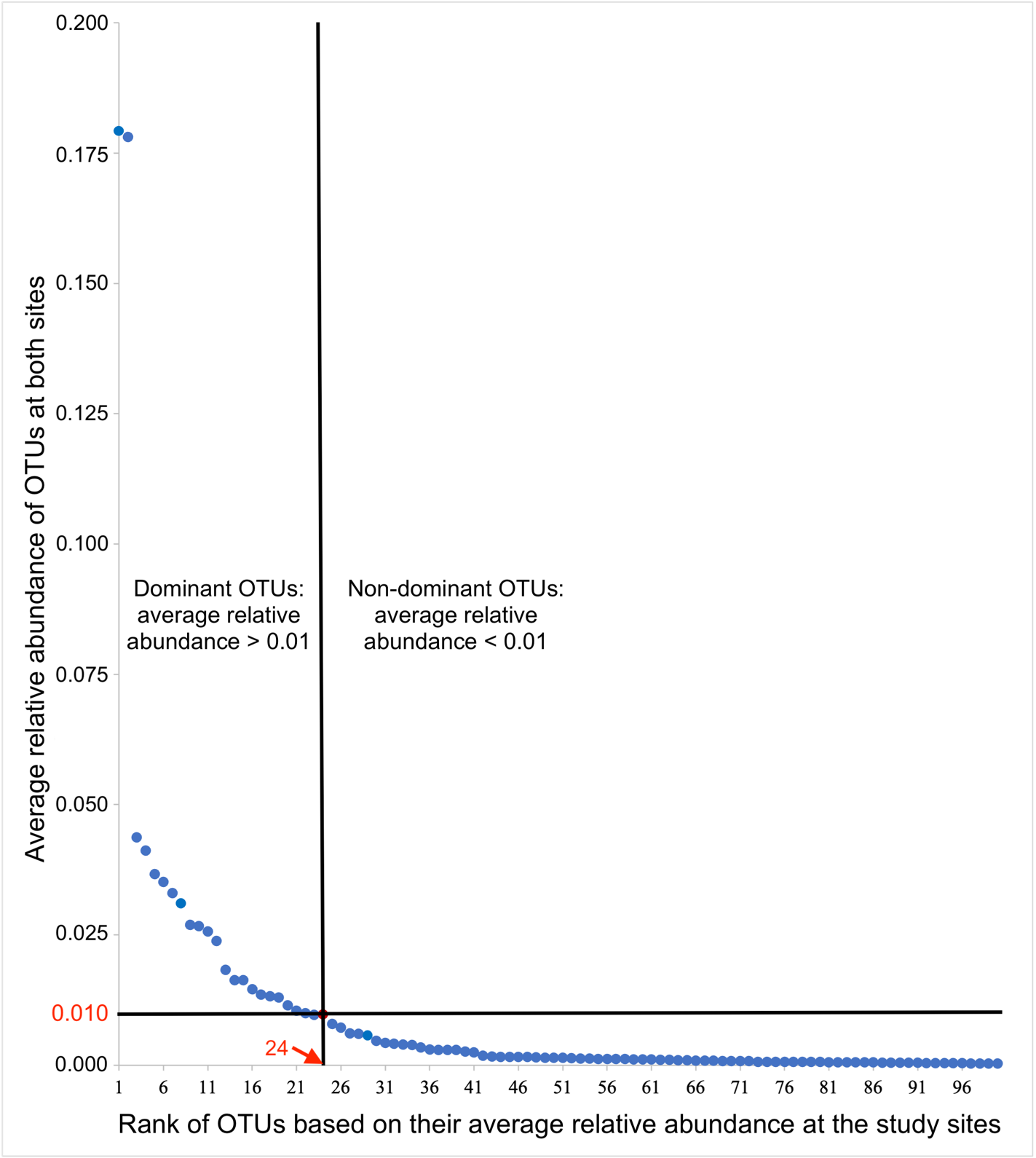


Online Resource 6 Demarcation of dominant OTUs from other satellite OTUs in the root and surrounding soil AMF communities associated with Cryptomeria japonica

AMF, arbuscular mycorrhizal fungi; OTUs, operational taxonomic units. The red point corresponds to rank 24, after which the difference between the cumulative relative abundance at two consecutive ranks is < 0.01.


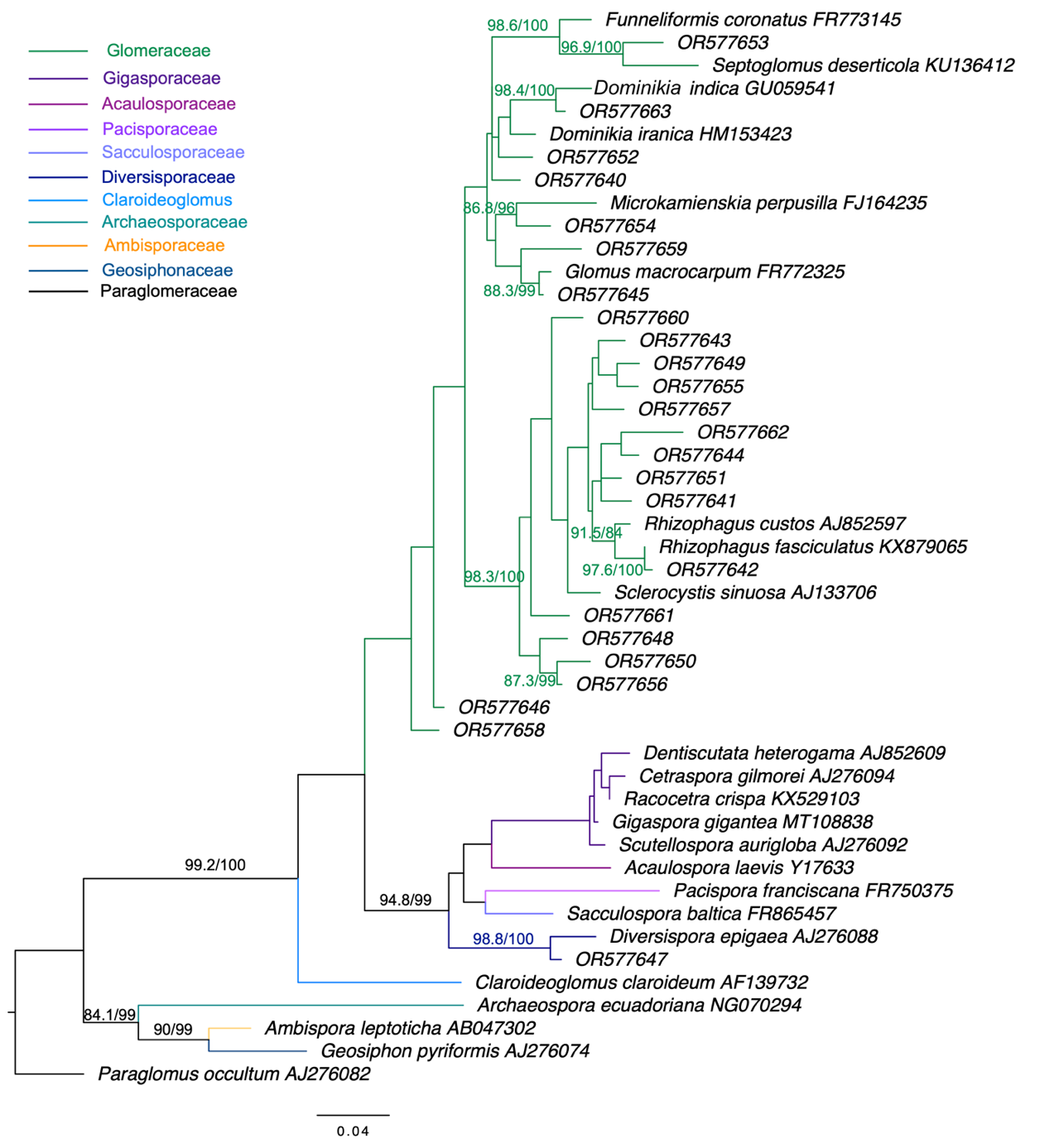


Online Resource 7 Phylogenetic tree of the dominant OTUs in the Cryptomeria japonica AMF communities investigated seasonally at two sites

AMF, arbuscular mycorrhizal fungi; OTUs, operational taxonomic units. The maximum likelihood tree was built using the representative sequences of the dominant core OTUs (24 nucleotide sequences) and 23 reference nucleotide sequences downloaded from the GenBank and MaarjAM databases. The best model and parameters were selected with the automatic model finder in IQ-TREE 2. The SH-aLRT test and ultrafast bootstrap (UFBoot) were performed with 1,000 randomizations. SH-aLRT values ≥ 80% and UFBoot values ≥ 95% are indicated at nodes. Dominant OTUs are labeled with accession numbers; reference sequences are labeled with scientific names followed by accession numbers. Aligned sequences covered 570 nucleotides of the small subunit ribosomal DNA between primers NS31 and AM1.


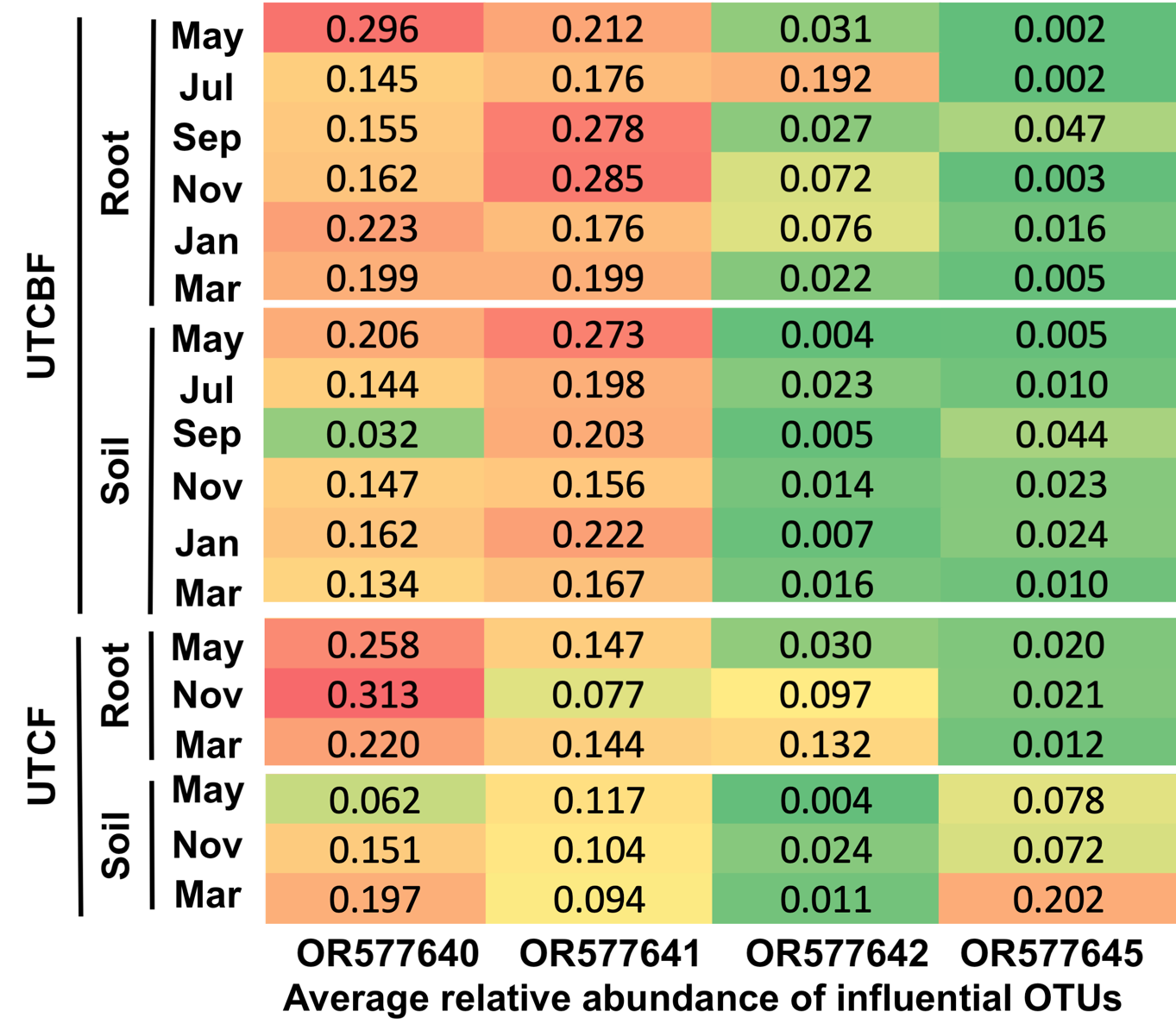


Online Resource 8 Relative abundance of the most influential dominant AMF OTUs in roots and surrounding soil of Cryptomeria japonica investigated seasonally at two sites in central Japan

AMF, arbuscular mycorrhizal fungi; OTUs, operational taxonomic units; UTCBF, University of Tokyo Chiba Forest; UTCF, University of Tokyo Chichibu Forest.
